# SILGGM: An extensive R package for efficient statistical inference in large-scale gene networks

**DOI:** 10.1371/journal.pcbi.1006369

**Published:** 2018-08-13

**Authors:** Rong Zhang, Zhao Ren, Wei Chen

**Affiliations:** 1 Department of Statistics, University of Pittsburgh, Pittsburgh, Pennsylvania, United States of America; 2 Division of Pulmonary Medicine; Department of Pediatrics, Children’s Hospital of Pittsburgh of UPMC, University of Pittsburgh, Pittsburgh, Pennsylvania, United States of America; 3 Department of Biostatistics, University of Pittsburgh Graduate School of Public Health, Pittsburgh, Pennsylvania, United States of America; bioinformatics, GERMANY

## Abstract

Gene co-expression network analysis is extremely useful in interpreting a complex biological process. The recent droplet-based single-cell technology is able to generate much larger gene expression data routinely with thousands of samples and tens of thousands of genes. To analyze such a large-scale gene-gene network, remarkable progress has been made in rigorous statistical inference of high-dimensional Gaussian graphical model (GGM). These approaches provide a formal confidence interval or a p-value rather than only a single point estimator for conditional dependence of a gene pair and are more desirable for identifying reliable gene networks. To promote their widespread use, we herein introduce an extensive and efficient R package named SILGGM (Statistical Inference of Large-scale Gaussian Graphical Model) that includes four main approaches in statistical inference of high-dimensional GGM. Unlike the existing tools, SILGGM provides statistically efficient inference on both individual gene pair and whole-scale gene pairs. It has a novel and consistent false discovery rate (FDR) procedure in all four methodologies. Based on the user-friendly design, it provides outputs compatible with multiple platforms for interactive network visualization. Furthermore, comparisons in simulation illustrate that SILGGM can accelerate the existing MATLAB implementation to several orders of magnitudes and further improve the speed of the already very efficient R package FastGGM. Testing results from the simulated data confirm the validity of all the approaches in SILGGM even in a very large-scale setting with the number of variables or genes to a ten thousand level. We have also applied our package to a novel single-cell RNA-seq data set with pan T cells. The results show that the approaches in SILGGM significantly outperform the conventional ones in a biological sense. The package is freely available via CRAN at https://cran.r-project.org/package=SILGGM.

This is a *PLOS Computational Biology* Software paper.

## Introduction

Gene co-expression network is an undirected graph, where each node represents a gene and each edge between two genes shows a significant co-expression relationship [1]. It has been of great biological interests and widely used in exploring underlying mechanisms of complex biological processes since the co-expressed genes are usually functionally related and share a pathway [2–5]. However, it is always a concern whether the inferred gene network structure is trustworthy or not. A partial correlation-based approach to assess the conditional dependence of two genes given the conditions of other genes in a network is a more reliable choice to infer a gene network since the marginal correlation may fail to reflect a true gene-gene relationship without considering other genes’ effects. Gaussian graphical model (GGM) is a typical statistical model to interpret gene dependence with the conditions of other genes.

Previous high-throughput sequencing technologies like microarray and bulk RNA-seq have generated many high-dimensional gene expression data sets with a huge number of genes, but these data sets usually have a small number of subjects or samples. Recently, the emergence of the droplet-based single-cell RNA-seq [6, 7] has made the cell-level gene measurements available, and its increasing availability has led to a growing number of even larger gene expression data sets which generally have thousands of subjects and tens of thousands of genes. These high-dimensional settings have imposed bigger statistical and computational challenges in obtaining a reliable gene network.

Due to the assumption of the intrinsically sparse structure of a gene network, two main streams of approaches have been developed in estimating conditional dependence of genes using high-dimensional GGM: (i.) the graphical Lasso, which is a penalized-likelihood approach for precision matrix of GGM [8–10] and (ii.) a neighbourhood-based approach with a penalized regression [11–13]. Over the recent three to four years, more important efforts have been made in rigorous statistical inference of gene-gene conditional dependence with high-dimensional GGM: the bivariate nodewise scaled Lasso (B_NW_SL) [14], the de-sparsified nodewise scaled Lasso (D-S_NW_SL) [15], the de-sparsified graphical Lasso (D-S_GL) [16] and the GGM estimation with false discovery rate (FDR) control using scaled Lasso or Lasso (GFC_SL or GFC_L) [17]. These approaches have two main advantages over the ones in sole estimation: (i). the obtained estimators of conditional dependence are more precise and asymptotically efficient with each variance equal to the inverse of Fisher information; (ii). the estimators are asymptotically normal under a minimal sparsity condition (e.g. the maximum node degree satisfies $s=o(\sqrt{n}/\log\left(p\right))$), so the corresponding confidence intervals or p-values are provided besides point estimators for identifying a more reliable gene network.

There are some existing software packages for gene co-expression network analysis. For example, the popular R package WGCNA [18] provides functions to construct a gene co-expression network based on the marginal correlations. In terms of the partial correlation-based approaches particularly for large-scale settings, glasso [9] and huge [19] are two widely adopted packages for fast estimation of gene-gene conditional dependence based on the high-dimensional GGM. More recent packages include FastCLIME [20], flare [21] and XMRF [22]. Unlike the marginal correlation-based approaches and high-dimensional GGM estimation, there are in practice few efficient packages or algorithms for the aforementioned approaches of rigorous statistical inference with the partial correlations that are supposed to be more powerful in large-scale gene-gene network analysis. FastGGM [23] is the recently developed package for an efficient and tuning-free implementation of B_NW_SL and has made the method computationally feasible to tens of thousands of genes. However, some redundant steps in the algorithm can be further improved and the outputs in only a matrix format make the package less friendly to users. Except FastGGM, no efficient R package has been proposed for the other above related works, and the expensive computation of naïve implementation also remains a challenge for these approaches.

To enhance the influence of these cutting-edge statistical inference works in practical usage and address the computational challenge in high-dimensional settings even with large sample sizes, we develop a more comprehensive package called SILGGM (**S**tatistical **I**nference of **L**arge-scale **G**aussian **G**raphical **M**odel) that includes B_NW_SL, D-S_NW_SL, D-S_GL and GFC_SL or GFC_L. SILGGM has significantly increased the efficiency of each approach using fast algorithms, the “Rcpp” library [24] and some additional optimizations. It also provides a consistent framework of statistically efficient inference on both individual gene pair and all gene pairs by extending the implementation of B_NW_SL, D-S_NW_SL and D-S_GL to global inference with FDR control under the framework of GFC_SL or GFC_L. Compared to FastGGM, SILGGM has several advantages. First, some steps in inner product calculations are optimized in the core algorithm of SILGGM, so B_NW_SL is performed even faster than its implementation in FastGGM. Second, SILGGM can accommodate users’ different research purposes with a new functionality of global inference for FDR control and with more flexible choices of methods. Third, based on users’ preference, the outputs in SILGGM can also be saved in a table format that is able to be further used directly in multiple platforms for network visualization like Cytoscape [25], BisoGenet [26] and BiNA [27]. Overall, the package SILGGM is an extensive and user-friendly tool that aims to facilitate large-scale gene network analysis with rigorous statistical inference and to show more trustworthy statistical results in a biological sense.

## Design and implementation

In GGM, a set of *p*-dimensional random variables *X* = (*X*_1_,*X*_2_,…,*X*_*p*_)′ follows a multivariate normal distribution with mean *μ* (assuming *μ* = 0 without loss of generality) and covariance matrix Σ. The conditional dependence between each pair of variables is reflected in a precision matrix Ω = (*ω*_*ij*_)_*p*×*p*_ = Σ^−1^, the inverse of Σ. For instance, if *X*_*i*_ and *X*_*j*_ are conditionally dependent, then equivalently, the corresponding element in Ω is *ω*_*ij*_ ≠ 0 [28]. In the gene network analysis, we regard *X*_*i*_ as the *i*^*th*^ gene. Therefore, the inference between gene *i* and *j* is equivalent to the inference of an individual *ω*_*ij*_, and the global inference of whole-scale gene pairs is based on a multiple testing procedure with all *ω*_*ij*_’s.

### Software architecture

**Fig 1:**
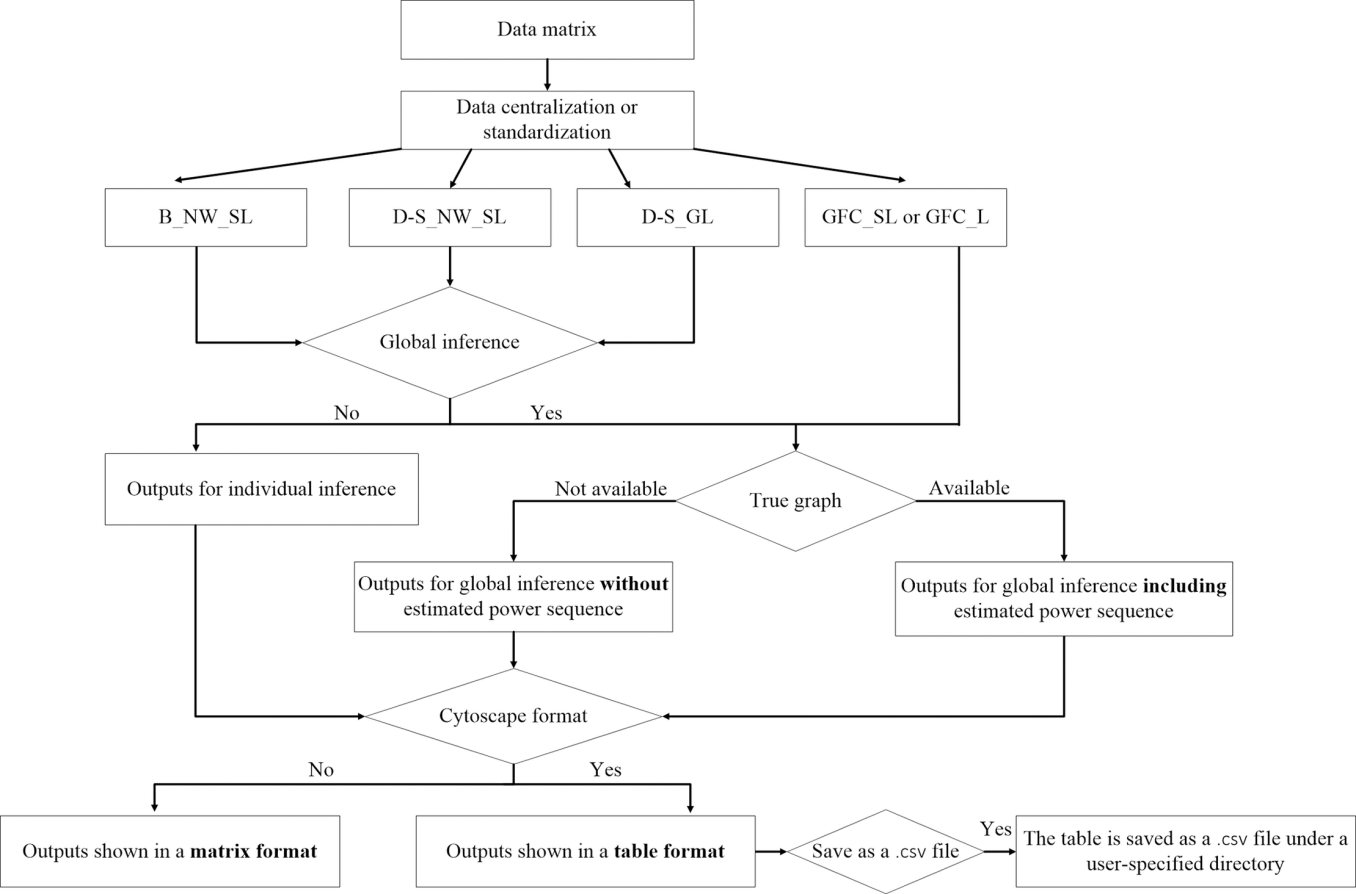
The workflow of the SILGGM package.

We focus on the high-dimensional settings with *p* (the number of genes) allowed to be far larger than *n* (the number of subjects). The SILGGM package has one main function SILGGM() with various arguments and its workflow is described in Fig 1.

The setup of the SILGGM() function is very simple. It only takes an *n* by *p* gene expression data matrix as an input. The gene names can be specified in each column by users. Without loss of generality, the data matrix is further centralized by subtracting its mean or standardized by subtracting the mean and adjusting the variance to one before the formal statistical inference, but the final results are returned in an original scale.

The method argument in the function SILGGM() supports four approaches in rigorous statistical inference: B_NW_SL, D-S_NW_SL, D-S_GL, GFC_SL or GFC_L. In the original four papers, the first three methods are developed for inference of each individual *ω*_*ij*_, while the last one is proposed particularly for simultaneous inference of all *ω*_*ij*_’s. All of the four methods (see more details in S1 Appendix) can be summarized into two steps. The first step involves a Lasso-type regularization approach. The graphical Lasso is performed in D-S_GL, while *O*(*p*) or *O*(*sp*) runs of nodewise Lasso-type regressions are conducted among the other three methods. The second step is to obtain (*p*^2^ − *p*)/2 test statistics: (i.) the estimators ${\hat{\omega }}_{ij}’s$ for B_NW_SL; (ii.) the de-sparsified estimators ${\overset{ˇ}{\omega }}_{ij}’s$ for D-S_NW_SL and D-S_GL; (iii.) the de-sparsified newly-constructed test statistics ${\hat{T}}_{ij}’s$ for GFC_SL or GFC_L, each of which is asymptotically efficient and normal at $\sqrt{n}$ rate under a minimal sparseness condition.

As it can be seen, GFC_SL or GFC_L essentially relies on asymptotically normal test statistics for testing on *ω*_*ij*_’s, so the implementations of the other three methods can also be extended to global inference under its FDR framework [17] that has been rigorously proved to be valid in high-dimensional settings. The global argument in the function determines whether or not to perform global inference in the other three methods. Since global inference needs FDR control, an *α*-level sequence with *α* = 0.05,0.1 is pre-specified by the alpha argument in the function and it can be customized by users with different values.

Outputs are shown with the different type of inference. For individual inference of gene *i* and *j*, SILGGM not only provides the estimator ${\hat{\omega }}_{ij}$ or ${\overset{ˇ}{\omega }}_{ij}$, but also obtains the associated confidence interval, z-score and p-value. Each output of gene *i* and *j* is encoded in the (*i*,*j*)^*th*^ element of a *p* by *p* symmetric matrix with diagonal elements equal to 0. For global inference with a pre-specified *α*-level sequence, SILGGM further returns the estimated FDR sequence based on ${\hat{T}}_{ij}’s$ or z-scores of ${\hat{\omega }}_{ij}’s$ or ${\overset{ˇ}{\omega }}_{ij}’s$, the corresponding threshold sequence for absolute values of test statistics and a series of decisions for conditional dependence between each gene pair (a list of *p* by *p* adjacency matrices with each off-diagonal element value of 1 = conditionally dependent or 0 = conditionally independent). If the true structure of a gene network is available (e.g. a simulation study or a real study with sufficient prior knowledge), SILGGM also includes the estimated power sequence with respect to the estimated FDR sequence. Users can input the true structure in a matrix format via the true_graph argument in the SILGGM() function.

**Fig 2:**
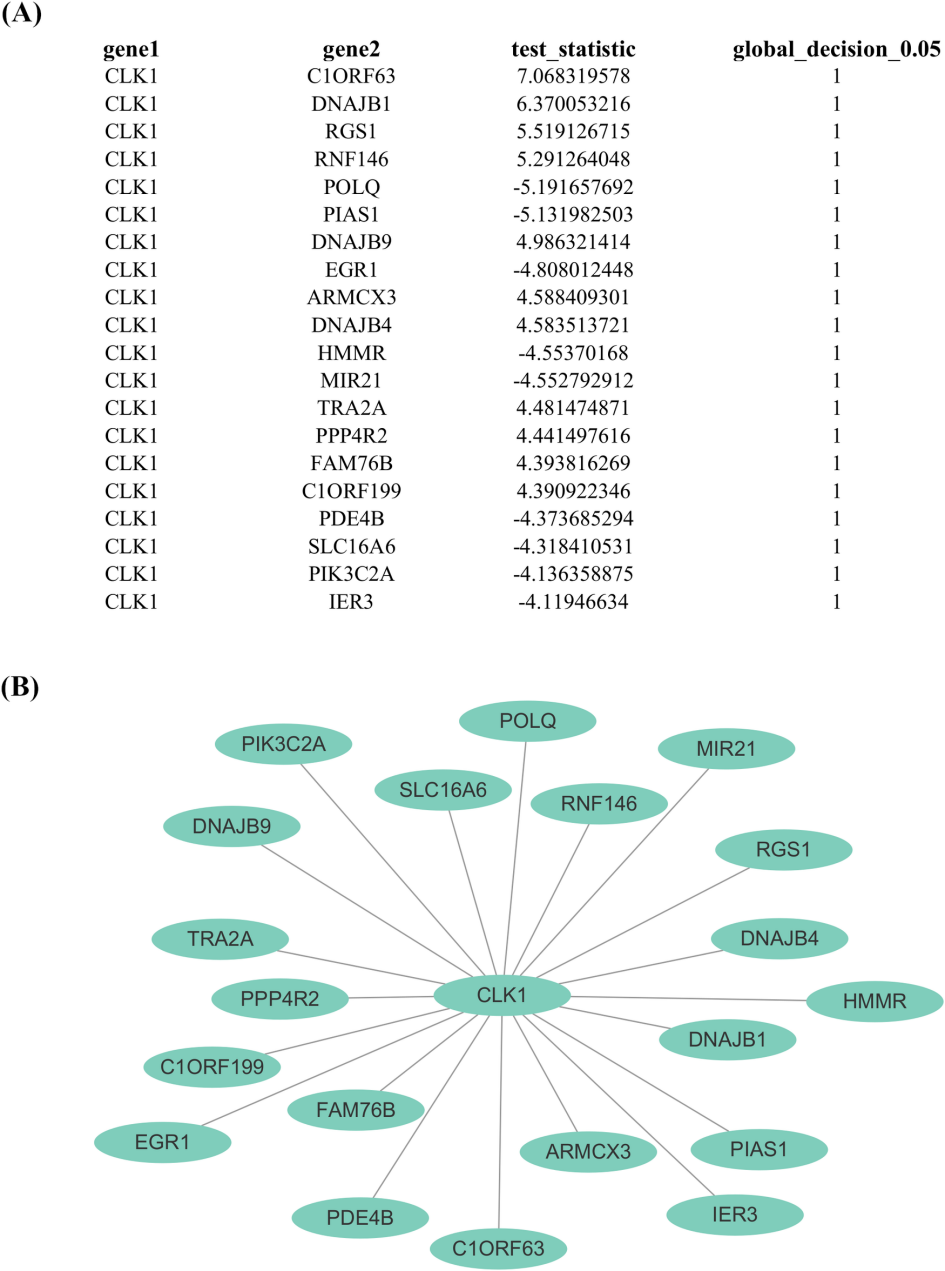
An example of table-format outputs and the corresponding network visualization. (A) A table in the .csv file generated by the SILGGM package using the method GFC_SL. (B) The corresponding network visualization.

In addition to present the above outputs from both individual and global inference in a matrix format, the function SILGGM() provides the cytoscape_format argument as an alternative to show them in a table format that can be saved as a .csv file by using the csv_save argument to a directory specified by the directory argument. The .csv file is compatible with multiple popular platforms for network visualization. In order to show the validity of this alternative, we have applied SILGGM to a public gene expression microarray data set on the lymphoblastoid cells of *n* = 258 asthmatic children [29, 30] and *p* = 1953 genes with the largest inter-sample variance (see “child_asthma.RData” in S2 Appendix) by using the method GFC_SL with FDR control at the level of 0.05. Fig 2 (A) gives a table in the .csv file with the 20 most significant gene pairs based on a rank of the absolute values of test statistics ${\hat{T}}_{ij}’s$ with the hub gene *CLK1* that has been proved to be susceptible to asthma [31]. The first two columns (“gene 1” and “gene 2”) show the names of each non-overlapped gene pair. The following column “test_statistic” indicates the test statistic ${\hat{T}}_{ij}$ of gene *i* and *j*. At the end, the column “global_decision_0.05” shows the decision for conditional dependence between each gene pair under global inference with FDR control at the 0.05 level. All the gene pairs are conditionally dependent in this example. Furthermore, we import the .csv file to Cytoscape (version 3.4.0) and obtain the corresponding network visualization shown in Fig 2 (B).

### Features of efficient implementations

Computational efficiency is a prominent advantage of SILGGM. The core algorithms in the package are developed with the “Rcpp” library [24] which highly speeds up the loop operation and makes the implementation of C++ code available in R. In addition to the fast programming language, there are many other key features of efficient implementations making SILGGM feasible in high-dimensional settings. We outline the details according to the two summarized steps of all the approaches as below.

In the first step, based on the same optimization in FastGGM [23], we pre-calculate and save the covariance matrix to avoid its repetitive calculation before solving each Lasso-type problem. Then, we apply the cyclical coordinate descent algorithm with covariance update [32] that has been shown much faster than other competing methods like the LARS procedure [33] in solving Lasso-type problems. To further increase the efficiency, some tuning-free schemes (e.g. the scaled Lasso with tuning parameter $\lambda =\sqrt{2\;\log\left(p/\sqrt{n}\right)/n}$, the graphical Lasso with a certain $\lambda =\sqrt{\log(p)/n}$ suggested in [16]) are applied to avoid the inefficient tuning selection. Our coding with the scaled Lasso is more efficient than directly using the package scalreg [13] which is built on the lars package [33]. To conduct the graphical Lasso in D-S_GL, we use the package glasso (version 1.8) [9] due to the great improvement in its efficiency by the screening procedures [34]. In addition, for GFC_L which requires tuning selection for FDR control, we apply the “warm start” optimizations [32] to boost the procedure.

In the second step, we facilitate inner product operations to derive each de-sparsified test statistic. To be more specific, we consider the sparsity of Lasso-type estimators from the first step and make inner product calculations only on the non-zero elements. For D-S_NW_SL or D-S_GL, to obtain ${\overset{ˇ}{\omega }}_{ij}$ needs an inner product which requires a total number of operations *O*(*p*^3^) with naïve calculation (see (7) and (10) in S1 Appendix). By considering the sparsity, the total number of operations can be reduced to *O*(*sp*^2^), and *s* is usually much smaller than *p* in high-dimensional settings.

In addition to the aforementioned optimizations, we optimize the inner product operations between the whole data matrix and regression coefficients by considering the sparsity of estimated coefficients when solving each scaled Lasso problem. The idea behind the optimization is same as the one used in the second step, and it reduces the redundant steps and enables B_NW_SL to perform even faster in SILGGM than in FastGGM.

## Results

**Fig 3:**
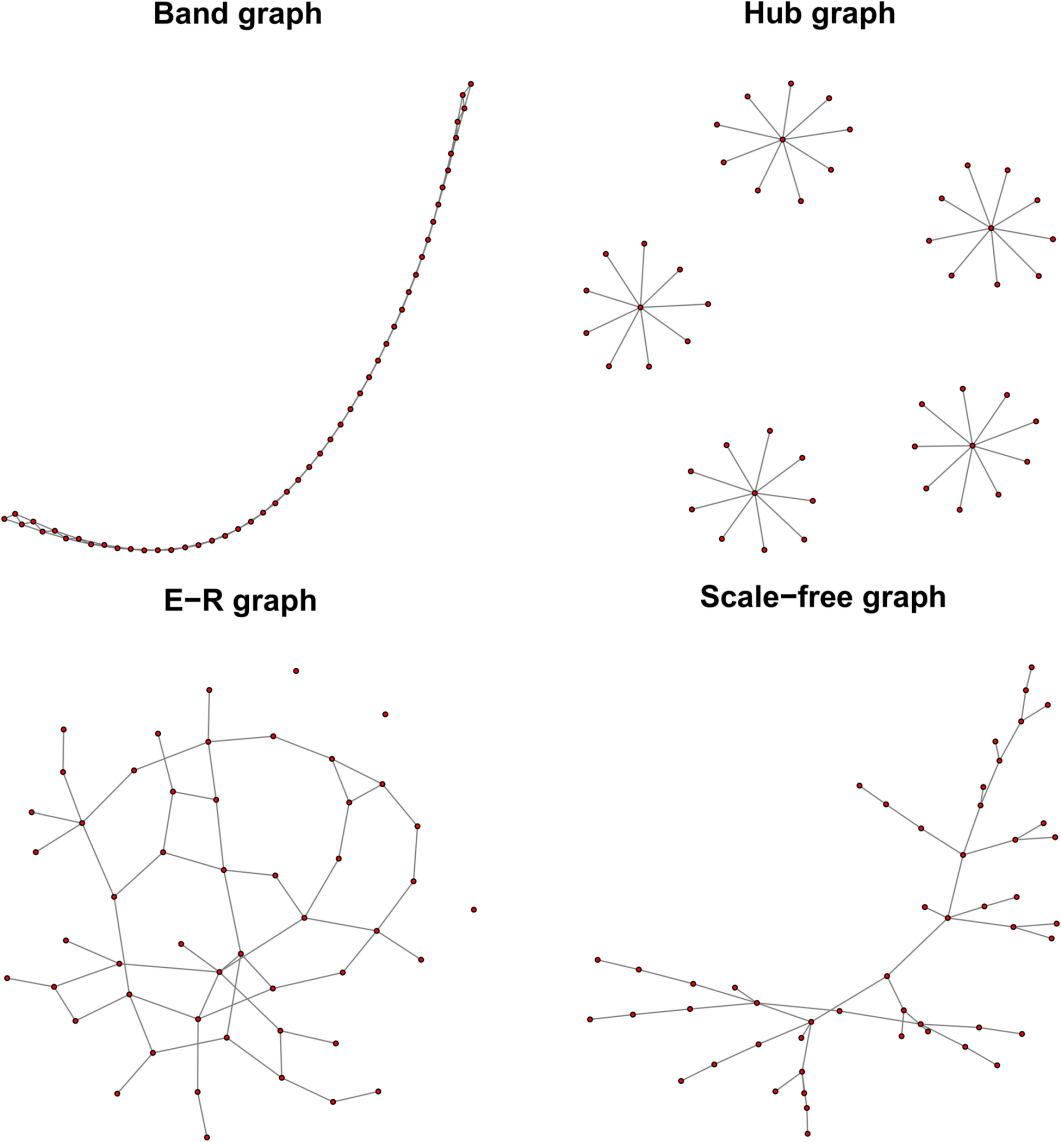
Four possible graph structures in simulation studies.

We illustrate the efficiency of SILGGM through simulation studies and a real data analysis. In simulation, we consider four popular graph structures for gene network studies: Band graph, Hub graph, Erdös–Rényi (E-R) random graph and Scale-free random graph, as shown in Fig 3 that is generated by the R package huge [19]. We not only evaluate time efficiency of SILGGM, but also make an extensive validation testing the estimation accuracy of SILGGM for both individual and global inference particularly in the very high-dimensional scenarios. Due to the limitation of space, more detailed results about individual inference and global inference are presented in S2 File and S3 File respectively. The real data analysis of SILGGM is based on a novel single-cell RNA-seq study on the gene expression of Pan T cells. All the R scripts are provided in S2 Appendix.

### Performance benchmark in simulation

To the best of our knowledge, the online MATLAB code (see “models.txt” and “GFC-lasso.txt” from http://math.sjtu.edu.cn/faculty/weidongl/Publication/code.rar in S2 Appendix) is the only publicly available implementation of GFC_L prior to the development of SILGGM. In order to compare its time performance with GFC_L implementation in SILGGM, we set *n* = 100 and simulate three types of graph settings: Band, Hub and E-R (see the details of the graph settings in S1 File), same as those in [17] with *p* = 50,100,200. Total timings (in seconds) over 100 replications on a single CPU are recorded for GFC_L with FDR control at the 0.1 and the 0.2 levels using SILGGM and the MATLAB code, as shown in Table 1. GFC_L implemented with SILGGM is generally around 60 times faster among all the scenarios and can be up to 70 times in some cases. The above simulations are conducted on a PC with Intel Core i5-3230M CPU @ 2.60GHz. The significant speed improvement in GFC_L implementation is mainly due to the incorporation of “Rcpp” library and the optimization of redundant steps of FDR calculation in tuning selection for FDR control.

**Table 1:** Total timings (in seconds) of GFC_L (SILGGM) and GFC_L (MATLAB).

	GFC_L (SILGGM)			GFC_L (MATLAB)		
***p***	50	100	200	50	100	200
**Band**	22.1	77.8	370.9	1396.2	4730.5	20039.3
**Hub**	22.8	84.1	363.7	1556.8	5227.7	19747.2
**E-R**	20.2	89.6	377.3	1495.7	5627.0	21482.2

Then, we evaluate the timing performance of B_NW_SL using SILGGM compared to the current package FastGGM. As shown in S1 Table, the E-R graph settings (see S1 File) same as those in [23] are simulated with *n* = 400,800 and *p* = 800,1000,2000,5000,10000 to make sure that the expected node degree of each graph, which is the value of *π* (the probability of *ω*_*ij*_ ≠ 0 for *i* ≠ *j*) times *p*, is around 4 or 5. The first column of S1 Table also gives the estimated average node degree of each case. We carry out the experiments on a Linux server with Intel Xeon CPU E5-2695 v2 @ 2.40GHz. To be as fair as possible, we perform B_NW_SL without global inference in SILGGM, so the outputs are same as the ones from FastGGM. Timings (in seconds) for one run on a single CPU with both SILGGM and FastGGM are reported in S1 Table using the same simulated data set from each graph setting. As it can be seen, B_NW_SL is implemented even faster in SILGGM among all the scenarios and the computational cost of each scenario is reduced by 20% ~ 56%.

In addition to the time evaluation, we validate the accuracy of estimation results from all the approaches for both individual and global inference in the very large-scale settings with relatively small sample sizes (*n* = 800, *p* = 5000 and *n* = 800, *p* = 10000).

We at first assess the performance of individual inference of each (*i*,*j*)^*th*^ gene pair (*H*_0_:*ω*_*ij*_ = 0 vs. *H*_1_:*ω*_*ij*_ ≠ 0) in terms of the estimation for an entire graph. The empirical Type I error (the probability of falsely rejecting *H*_0_ when there is actually a known zero partial correlation between gene *i* and *j*) under a pre-specified level of 0.05 for p-values and the corresponding Type II error (the probability of failing to reject *H*_1_ when there is actually a known non-zero partial correlation between gene *i* and *j*) are measured for Band graph (same as that described in [17]), E-R graph (same as that described in [17]) and Scale-free graph (see S2 File). The good performance of the empirical Type I and Type II error rates has shown the validity of all the approaches in the package SILGGM for individual inference even in the very high-dimensional scenarios (see more detailed results in S2 File). To make a further comparison for individual inference, we also evaluate the average empirical coverage probabilities for the 95% confidence intervals of the *ω*_*ij*_’s for the “non-zero partial correlation” set (a set of all pairs with non-zero *ω*_*ij*_’s) and the “zero partial correlation” set (a set of all pairs with zero *ω*_*ij*_’s) respectively. Since GFC_SL or GFC_L provides no confidence intervals, we involve the other three approaches here. According to the results from the three graph settings, the overall performance of the confidence intervals among B_NW_SL, D-S_NW_SL and D-S_GL are good in terms of the entire graph structure. But in terms of the confidence intervals for the non-zero partial correlations, B_NW_SL and D-S_NW_SL outperform D-S_GL. Moreover, the performance of B_NW_SL is more stable than that of D-S_NW_SL in the different settings (see more details in S2 File). Therefore, for individual inference of a gene pair which further requires the information of a confidence interval, B_NW_SL is a more desirable choice compared to the other approaches, but D-S_NW_SL can be an alternative to save time for the very high-dimensional cases.

Then, we evaluate the performance of global inference of all gene pairs for the overall partial correlation recovery in the very large-scale settings based on the same three graph settings (Band, E-R and Scale-free) used in individual inference. Unlike individual inference, global inference generally requires a multiple testing procedure for tests on all *H*_0_:*ω*_*ij*_ = 0 vs. *H*_1_:*ω*_*ij*_ ≠ 0 with 1 ≤ *i* < *j* ≤ *p* simultaneously. Therefore, to make global inference of all gene pairs in a large graph, we always recommend controlling FDR to avoid the inflation of false positives. The testing results from the three graph settings indicate that the FDRs of all the methods are effectively controlled below the desired level for both *p* = 5000 and *p* = 10000. The corresponding power values (the proportions of the correctly identified elements among the known non-zero partial correlations) and the Matthews correlation coefficients (MCCs) also demonstrate comparably good performance of all the methods (see S3 File for more details). Overall speaking, the good performance of FDR, power and MCC has shown the validity of all the approaches in correctly identifying the zero and the non-zero partial correlations in a global sense even for the very high-dimensional scenarios.

### Gene network analysis in a droplet-based single-cell data set with pan T cells

We have applied the SILGGM package to a novel public single-cell RNA-seq data set with pan T cells isolated from peripheral blood mononuclear cells of a healthy human donor. The data set generated by the latest CellRanger pipeline [35] includes *n* = 3555 cells. After filtering out the unexpressed genes, we consider *p* = 2000 genes with the largest inter-sample variance (see “sc_pan_T.RData” in S2 Appendix).

Since the genes in the data set are measured with the unique molecular identifier (UMI) counts [36], we need to transform the count values before the use of SILGGM. According to [37], it is reasonable to take a log2(UMI counts + 1) transformation and to perform a nonparanormal transformation [38] using the function huge.npn() in the package huge on the continuized data to make it Gaussian because the transformation procedure preserves the underlying network structure. Then, we perform each approach in SILGGM under global inference with FDR control at the 0.01 level. As comparison studies, we have also applied the graphical Lasso (GLasso) using the package huge, the marginal correlation-based approach with the Pearson’s correlation (PearsonCorr) and the maximum likelihood estimation (MLE) of the partial correlation by directly inverting sample covariance matrix to the same transformed data set. GLasso is run with the default parameters except using the rotational information criterion [19, 39] for tuning selection. Since GLasso only provides point estimates, a non-zero partial-correlation estimate here implies a conditional dependence between the gene pair. For PearsonCorr and MLE, we do need the same thresholding procedure used among the other approaches in SILGGM to control FDR at the 0.01 level based on the z-scores of the Fisher z-transformation of Pearson’s correlation and the z-scores of MLEs on all the gene pairs.

**Fig 4:**
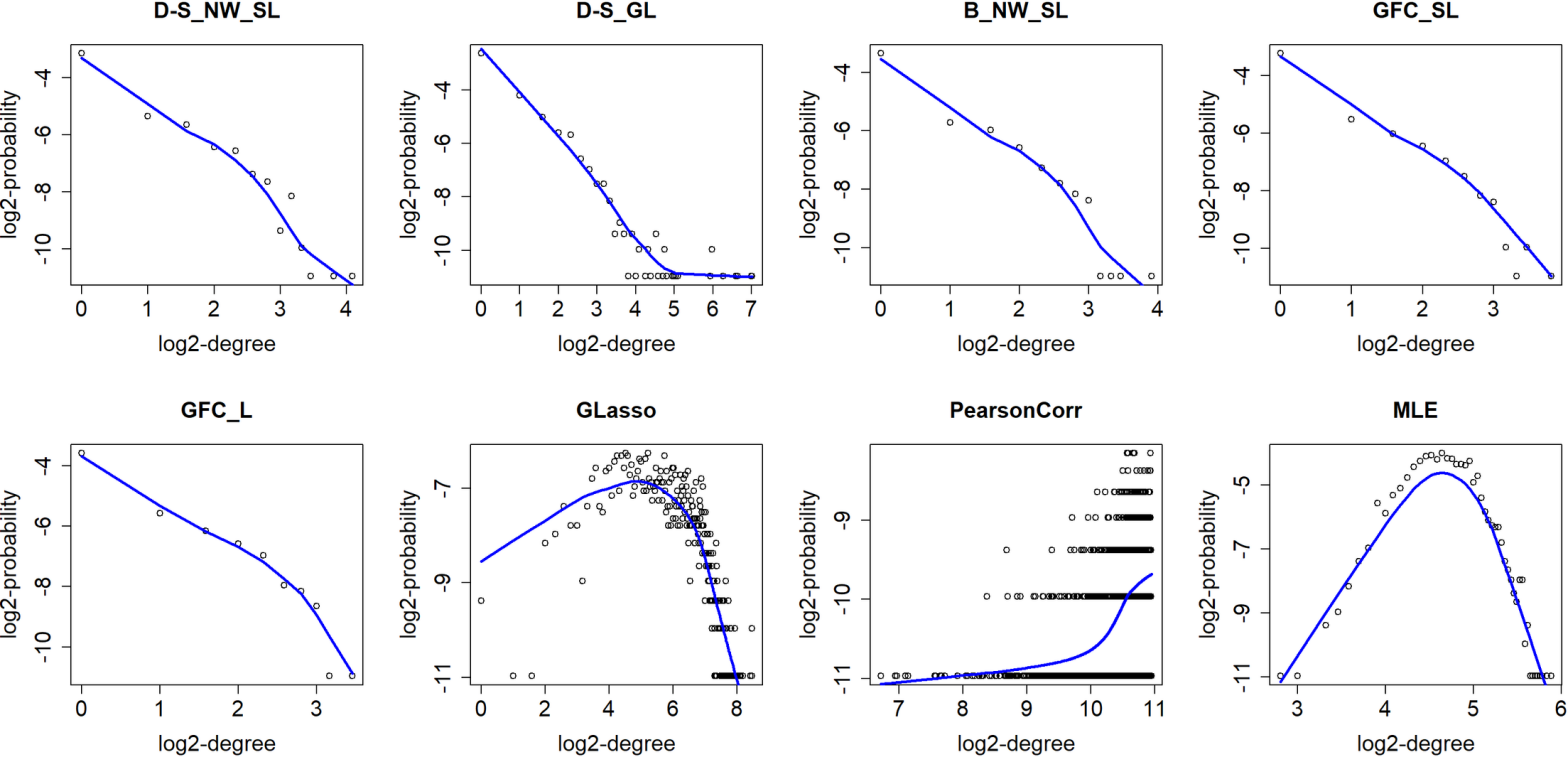
The log2-log2 plots of degree distribution of inferred networks by the different approaches.

Motivated by [37], we apply the power law [40, 41] to evaluate the performance of the overall network structure inferred by the different approaches. According to the power law, we have *p*(*m*) ∝ *m*^−*λ*^ for some positive *λ*. Here, *m* refers to the node degree and *p*(*m*) denotes the probability of the *m*-degree nodes. Many studies have indicated that biological networks are scale-free, and the node degrees possess a power-law distribution [42–45]. The log2-log2 plots of degree distribution of inferred networks are shown in Fig 4, where the blue curves are fitted by the R function lowess(). All the approaches in our package SILGGM fit the power-law relationship well, but Glasso, PearsonCorr and MLE do not. Even if *n* > *p* in this data set, the values of *n* and *p* share the same order such that MLE becomes unstable and increases bias of estimation. Thus, all the inferred network structures by SILGGM are biologically meaningful and much more reliable. Furthermore, we can see that the performance of the other three methods based on the nodewise Lasso-type regressions in SILGGM is even better than that of D-S_GL since the plot of D-S_GL shows some noise in the tail.

## Availability and future directions

The source code of the package and a complete reference manual including dependencies, usage of all package functions and associated examples are freely available via CRAN at https://cran.r-project.org/package=SILGGM. The details of package installation are described in S3 Appendix.

The package SILGGM is computationally efficient compared to the MATLAB implementation of GFC_L and the R package FastGGM. Since R is a publicly free platform and has been more widely used in biological research compared to MATLAB which is a piece of commercially licensed software and has less accessibility to biologists, the R platform-based SILGGM will play a more important role in accelerating the biological gene network studies. SILGGM is also statistically efficient with both individual and global inference due to the theoretical justification of the four approaches and the validation of estimation accuracy in simulation studies. The analytical results from the single-cell data with Pan T cells further reflect the statistical efficiency of SILGGM since inferred gene networks are more reliable. Moreover, the comprehensiveness of SILGGM allows users to have more flexible choices of methods depending on the specific purpose of their study. Due to its computational feasibility, analytical reliability in results and methodological comprehensiveness, SILGGM can become a valuable and powerful tool to a wide range of biological researchers for high-dimensional or even whole genome-wide co-expression network analysis.

In practice, users have flexible options on the approaches provided by SILGGM with respect to the specific purpose of their study. In a whole genome-wide study which is based on global inference of all gene pairs, GFC_L is the one we recommend when *n* is small (e.g. *n* = 100) because the tuning selection in GFC_L is beneficial for FDR control. When *n* becomes larger (e.g. *n* = 800) but may be still relatively small to *p*, users can choose any of the four approaches due to their similar performance among the different settings (see S3 File for more details). If the study purpose is to evaluate a small set of genes such as certain gene pathways that contribute to an important biological mechanism or a particular gene such as a hub gene that is closely related to a specific disease, among the inference results from all gene pairs, we recommend users choosing B_NW_SL since it provides confidence intervals in addition to p-values and its performance of confidence intervals is always good in the different settings (see S2 File for detailed comparisons). In a very large-scale setting with *p* increased to a ten thousand level, D-S_NW_SL is an alternative to save running time. Alternatively, if only the information of p-values is needed, we also recommend GFC_SL or GFC_L.

Besides high-dimensional microarray and bulk RNA-seq data, we intend to promote the application of SILGGM to single-cell RNA-seq data with both large *n* and *p*. The data sets from single-cell RNA-seq have substantial advantages over the ones from population-level microarray or bulk RNA-seq for us to explore the structure of a gene co-expression network due to larger sample sizes [6] and inherent cell-to-cell variability. According to [46], the gene network from a single-cell study is able to further reveal potential functionally-related gene pairs which are masked from the bulk sequencing.

In the future, we will add parallel computing to SILGGM so as to allow users to use multiple clusters for bigger data analysis since the droplet-based single-cell technology will further increase the sample size [6]. In addition, the new feature for the rigorous statistical inference of high-dimensional multiple gene networks is another potential extension of our package because differential gene network analysis among different cell types or cells of multiple individuals is being paid more attention to.

## Supporting information

S1 Appendix Theoretical procedures of each method included in the package SILGGM. (PDF)

S2 Appendix MATLAB code, R scripts and related data sets for all examples, simulation studies and real data analysis. (RAR)

S3 Appendix The package installation. (PDF)

S1 File The graph settings for time evaluation in simulation studies. (PDF)

S2 File Testing on the accuracy of individual inference. (PDF)

S3 File Testing on the accuracy of global inference. (PDF)

S1 Table Timings (in seconds) of B_NW_SL (SILGGM) and B_NW_SL (FastGGM). (PDF)
