# Supplementary material for: SILGGM: An extensive R package for efficient statistical inference in large-scale gene networks
Source: PLoS Comput Biol. 2018 Aug 13;14(8):e1006369. doi: 10.1371/journal.pcbi.1006369 (PMC6107288; doi:10.1371/journal.pcbi.1006369)
Supplement: S1 Appendix — (PDF) [file pcbi.1006369.s001.pdf]

S1 Appendix. Theoretical procedures of each method included in the package SILGGM

Without loss of generality, we assume that $\boldsymbol{X} = (X_1, \ldots, X_p)$ is an $n \times p$ matrix, where each row vector $(X_{k1}, \ldots, X_{kp})'$ for $1 \leq k \leq n$ follows a p-dimensional independently and identically multivariate normal distribution with mean 0 and covariance matrix Σ. The precision matrix is denoted as $\Omega = (\omega_{ij}) = \Sigma^{-1}$, where $i, j = 1,2, \ldots p$.

1. The bivariate nodewise scaled Lasso (B_NW_SL)

B_NW_SL [1] is to make inference on each ω_{ij} with $i \neq j$. Based on the bivariate conditional normal distribution with index set $A = \{i, j\}$,

$$\boldsymbol{X}_A | \boldsymbol{X}_{A^c} \sim N\left(-\Omega_{A,A}^{-1}\Omega_{A,A^c}\boldsymbol{X}_{A^c}, \Omega_{A,A}^{-1}\right),\ \Omega_{A,A} = \begin{pmatrix} \omega_{ii} & \omega_{ij} \\ \omega_{ji} & \omega_{jj} \end{pmatrix}, \quad (1)$$

the bivariate nodewise scaled Lasso regression of the two variables in A against the other variables in A^c is proposed,

$$\operatorname{argmin}_{\beta \in R^{p-2}, \sigma \in R^+} \left\{ \frac{\|\boldsymbol{X}_m - \boldsymbol{X}_{A^c}\beta\|^2}{2n\sigma} + \frac{\sigma}{2} + \lambda \sum_{k \in A^c} \frac{\|X_k\|}{\sqrt{n}} |\beta_k| \right\},\ m \in A = \{i, j\}. \quad (2)$$

Here, each run of scaled Lasso regression is tuning-free and the tuning parameter is taken as $\lambda = \sqrt{2\log(p/\sqrt{n})/n}$. The estimated residual $\hat{\varepsilon}_A = \boldsymbol{X}_A - \boldsymbol{X}_{A^c}\hat{\beta}_A$ can be obtained once $\hat{\beta}_A$ is estimated from (2). Then, Ω for variables i and j can be estimated:

$$\widehat{\Omega}_{A,A} = \begin{pmatrix} \widehat{\omega}_{ii} & \widehat{\omega}_{ij} \\ \widehat{\omega}_{ij} & \widehat{\omega}_{jj} \end{pmatrix} = \left(\frac{1}{n}\hat{\varepsilon}_A'\hat{\varepsilon}_A\right)^{-1},\ A = \{i, j\}. \quad (3)$$

Under the minimal sparseness assumption $s = o(\sqrt{n}/\log(p))$, each estimator $\widehat{\omega}_{ij}$ has been shown asymptotically normal and efficient,

$$\sqrt{n\left(\widehat{\omega}_{ii}\widehat{\omega}_{jj} + \widehat{\omega}_{ij}^2\right)^{-1}}\left(\widehat{\omega}_{ij} - \omega_{ij}\right) \xrightarrow{D} N(0,1). \quad (4)$$

According to (4), we can estimate the corresponding p-value and confidence interval of each ω_{ij}. The naive implementation of the procedure requires $O(p^2)$ runs of scaled Lasso regression, but the total number of runs of regression can be reduced to $O(sp)$ in terms of the comments in [1] and the implementation in [2].

2. The de-sparsified nodewise scaled Lasso (D-S_NW_SL)

D-S_NW_SL [3] is based on the p runs of nodewise scaled Lasso regression for i^{th} variable against all the other variables i^c,

$$\text{argmin}_{\beta_i \in R^{p-1}, \sigma \in R^+} \left\{ \frac{\|X_i - \boldsymbol{X}_{i^c}\beta_i\|^2}{2n\sigma} + \frac{\sigma}{2} + \lambda \sum_{k \in i^c} |\beta_{ik}| \right\}. \quad (5)$$

Again, the tuning parameter for each run of regression is taken as $\lambda = \sqrt{2\log(p/\sqrt{n})/n}$. Unlike the previous B_NW_SL, the procedure deals with the coefficients rather than the regression noise. If $\hat{\beta}_i$'s are the estimated coefficients from (5), we can define $\hat{\sigma}_i^2 = \|X_i - \boldsymbol{X}_{i^c}\hat{\beta}_i\|^2/n$, $\tilde{\sigma}_i^2 = \hat{\sigma}_i^2 + \lambda\hat{\sigma}_i\|\hat{\beta}_i\|_1$ and $\hat{B}_i = \left(-\hat{\beta}_{i,1}, \ \ldots, \ -\hat{\beta}_{i,i-1}, \ 1, \ -\hat{\beta}_{i,i+1}, \ldots, -\hat{\beta}_{i,p}\right)^T$. Then, the ith column of Ω can be estimated:

$$\widehat{\omega}_i = \hat{B}_i/\tilde{\sigma}_i^2. \quad (6)$$

However, it is well known that the initial estimators in (6) have bias, so the authors have proposed a bias correction procedure on $\widehat{\omega}_{ij}$. Under the Karush-Kuhn-Tucker (KKT) conditions, the de-sparsified (or de-biased) estimator $\breve{\omega}_{ij}$ is

$$\breve{\omega}_{ij} = \widehat{\omega}_{ij} + \widehat{\omega}_{ji} - \widehat{\omega}_i^T \hat{\Sigma} \widehat{\omega}_j, \quad (7)$$

where $\hat{\Sigma} = \boldsymbol{X}^T\boldsymbol{X}/n$.

Under the minimal sparseness assumption $s = o(\sqrt{n}/\log(p))$, each de-biased estimator $\breve{\omega}_{ij}$ has achieved the asymptotically efficient result with

$$\sqrt{n\left(\widehat{\omega}_{ii}\widehat{\omega}_{jj} + \widehat{\omega}_{ij}^2\right)^{-1}}\left(\breve{\omega}_{ij} - \omega_{ij}\right) \xrightarrow{D} N(0,1). \tag{8}$$

According to (8), the corresponding p-value and confidence interval can be estimated for each ω_{ij}.

3. The de-sparsified graphical Lasso (D-S_GL)

D-S_GL [4] also depends on a bias correction procedure which is very similar to the one in D-S_NW_SL. However, the initial estimator $\widehat{\Omega} = (\widehat{\omega}_{ij})_{p\times p}$ here is obtained by solving a graphical Lasso optimization problem for Ω:

$$\operatorname{argmin}_{\Omega}\left\{\operatorname{trace}\left(\Omega^T\widehat{\Sigma}\right) - \log\det(\Omega) + \lambda\|\Omega\|_{1,\text{off}}\right\}. \tag{9}$$

Even though (9) is not tuning-free, the tuning parameter can be taken as $\lambda = \sqrt{\log(p)/n}$ according to the suggestion in [4]. Then, with the same idea of bias correction in D-S_NW_SL, the de-sparsified (or de-biased) estimator $\breve{\Omega} = (\breve{\omega}_{ij})_{p\times p}$ is

$$\breve{\Omega} = 2\widehat{\Omega} - \widehat{\Omega}\widehat{\Sigma}\widehat{\Omega}. \tag{10}$$

Under the minimal sparseness assumption $s = o(\sqrt{n}/\log(p))$, each de-biased estimator $\breve{\omega}_{ij}$ achieves the same asymptotically efficient result as the one in (8).

4. The Gaussian graphical model (GGM) estimation with false discovery rate (FDR) control using scaled Lasso or Lasso (GFC_SL or GFC_L)

While the previous three methods are originally developed for individual inference of each ω_{ij}, GFC_SL or GFC_L [5] is proposed particularly for global inference of all ω_{ij}'s. The approach is based on a bias correction procedure on the sample covariance of residuals between

each pair of variables i and j. In order to obtain the estimators of residuals, the first step of the method needs p runs of nodewise scaled Lasso regression same as those in (5) or nodewise Lasso regression for i^{th} variable against all the other variables i^c as below,

$$\operatorname{argmin}_{\beta_i \in R^{p-1}} \left\{ \frac{\|X_i - \boldsymbol{X}_{i^c}\beta_i\|^2}{2n} + \lambda_i \sum_{k \in i^c} |\beta_{ik}| \right\}. \quad (11)$$

If the estimated coefficients $\hat{\beta}_i$'s are obtained from (5) or (11), then we can obtain the estimated residual $\hat{\varepsilon}_i = X_i - \boldsymbol{X}_{i^c}\hat{\beta}_i$ and the estimated sample covariance of residuals between $(i,j)^{th}$ pair of variables $\hat{r}_{ij} = \frac{1}{n}\sum_{k=1}^{n} \hat{\varepsilon}_{ki}\hat{\varepsilon}_{kj}$. The second step is to make a bias correction on $\hat{r}_{ij}$ to obtain

$$T_{ij} = \frac{1}{n}\left(\sum_{k=1}^{n} \hat{\varepsilon}_{ki}\hat{\varepsilon}_{kj} + \sum_{k=1}^{n} \hat{\varepsilon}_{ki}{}^2 \hat{\beta}_{ji} + \sum_{k=1}^{n} \hat{\varepsilon}_{kj}{}^2 \hat{\beta}_{i(j-1)}\right),\ 1 \le i < j \le p \quad (12)$$

and construct a new test statistic

$$\hat{T}_{ij} = \sqrt{\frac{n}{\hat{r}_{ii}\hat{r}_{jj}}}\, T_{ij} \quad (13)$$

for the multiple testing

$$H_0\colon \omega_{ij} = 0 \quad \text{vs.} \quad H_1\colon \omega_{ij} \neq 0. \quad (14)$$

Under the null hypothesis in (14) and the same minimal sparseness assumption as before, (13) has an asymptotically normal result with

$$\hat{T}_{ij} \xrightarrow{D} N(0,1). \quad (15)$$

Since GFC_SL or GFC_L is developed for global inference, another main component of this method is to provide a novel framework for FDR control that has been theoretically proved valid in high-dimensional settings. It is well known that the false discovery proportion (FDP) with a threshold t can be written as

$$\mathrm{FDP}(t) = \sum_{(i,j)\in H_0} I\{|\hat{T}_{ij}| \ge t\} / \max\{\sum_{1 \le i < j \le p} I\{|\hat{T}_{ij}| \ge t\},\ 1\}. \quad (16)$$

To control FDR needs to control (16) since we have $\mathrm{E}\big(\mathrm{FDP}(t)\big) = \mathrm{FDR}(t)$. The numerator of (16) is generally unknown, but according to [5], the author has proved that

$$\sum_{(i,j)\in H_0} I\{|\hat{T}_{ij}| \geq t\} \approx 2(1-\Phi(t))(p^2-p)/2, \tag{17}$$

where $\Phi(\cdot)$ is a standard normal cumulative distribution function. Therefore, we can choose the threshold

$$\hat{t} = \inf\left\{0 \leq t \leq 2\sqrt{\log(p)}:\ \frac{2(1-\Phi(t))(p^2-p)/2}{\max\{\sum_{1\leq i<j\leq p} I\{|\hat{T}_{ij}|\geq t\}, 1\}} \leq \alpha\right\},\quad 0 \leq \alpha \leq 1. \tag{18}$$

We reject H_0 in (14) if $|\hat{T}_{ij}| \geq \hat{t}$.

As an alternative to the tuning-free scaled Lasso regression, each run of (11) for X_i against $\boldsymbol{X}_{i^c}$ requires a selection of the tuning parameter $\lambda_i = \delta\sqrt{\hat{\sigma}_{ii}^2 \log(p)\,/\,n}$, where $\hat{\sigma}_{ii}^2 = \sum_{k=1}^{n} {X_{ki}}^2\,/n$, with a data-driven choice of δ from 0 to 2. The following data-driven scheme is used based on the result in (17):

$$\delta = \hat{l}/N,$$

$$\hat{l} = \operatorname{argmin}_{0\leq l\leq 2N} \sum_{m=3}^{9} \left(\frac{\sum_{1\leq i\neq j\leq p} I\{|\hat{T}_{ij}(l/N)|\geq \Phi^{-1}(1-k/20)\}}{k(p^2-p)/10} - 1\right)^2. \tag{19}$$

Here, $N = 20$ is set by default and a different value of N can be set up in practice.

Since the previous three methods have asymptotically normal results in terms of (4) and (8), the FDR framework described in (17) and (18) can also be applied to them by replacing $\hat{T}_{ij}$ with a different test statistic based on $\widehat{\omega}_{ij}$ or $\breve{\omega}_{ij}$. Therefore, the implementations of B_NW_SL, D-S_NW_SL and D-S_GL are allowed for global inference as well.

References

1. Ren Z, Sun T, Zhang CH, Zhou HH. Asymptotic normality and optimalities in estimation of large Gaussian graphical models. Ann Stat. 2015; 43: 991-1026.

2. Wang T, Ren Z, Ding Y, Fang Z, Sun Z, Macdonald ML, et al. FastGGM: an efficient algorithm for the inference of gaussian graphical model in biological networks. PLoS Comput Biol. 2016; 12: e1004755.
3. Janková J, Van de Geer S. Honest confidence regions and optimality in high-dimensional precision matrix estimation. Test. 2017; 26: 143-162.
4. Janková J, Van de Geer S. Confidence intervals for high-dimensional inverse covariance estimation. Electron J Stat. 2015; 9: 1205-1229.
5. Liu W. Gaussian graphical model estimation with false discovery rate control. Ann Stat. 2013; 41: 2948-2978.
