# Supplementary material for: SILGGM: An extensive R package for efficient statistical inference in large-scale gene networks
Source: PLoS Comput Biol. 2018 Aug 13;14(8):e1006369. doi: 10.1371/journal.pcbi.1006369 (PMC6107288; doi:10.1371/journal.pcbi.1006369)
Supplement: S3 Appendix — (PDF) [file pcbi.1006369.s003.pdf]

S3 Appendix. The package installation

- Windows users should install “Rtools” before installation of this package.
- The package SILGGM is available on CRAN and can be installed using the following R commands:

  install.packages(“Rcpp”)

  install.packages(“SILGGM”)

  The first line can be omitted if “install dependencies” is checked in the R package installer.

- When the source code file “SILGGM_1.0.0.tar.gz” is downloaded from CRAN, the package can also be installed:

  install.packages(“Rcpp”)

  install.packages(pkgs = “SILGGM_1.0.0.tar.gz”, repos = NULL, type = “source”)
