# Supplementary material for: SILGGM: An extensive R package for efficient statistical inference in large-scale gene networks
Source: PLoS Comput Biol. 2018 Aug 13;14(8):e1006369. doi: 10.1371/journal.pcbi.1006369 (PMC6107288; doi:10.1371/journal.pcbi.1006369)
Supplement: S1 File — (PDF) [file pcbi.1006369.s004.pdf]

S1 File. The graph settings for time evaluation in simulation studies

1. The graph settings for time evaluation on the implementation of GFC_L

- **Band graph:** a p by p precision matrix $\Omega = (\omega_{ij})_{p\times p}$ with $\omega_{i,i+1} = \omega_{i+1,i} = 0.6$, $\omega_{i,i+2} = \omega_{i+2,i} = 0.3$ and the other off-diagonal elements $\omega_{ij} = 0$ for $|i-j| \geq 3$. The diagonal entries of Ω are $\omega_{ii} = 1$ for $i = 1,2,3\ldots,p$. The expected node degree of the graph is 4.
- **Hub graph:** an initial p by p matrix $\Omega' = (\omega_{ij})_{p\times p}$ with $\omega_{ij} = \omega_{ji} = 0.5$ for $i = 10(r-1)+1$, $10(r-1)+2 \leq j \leq 10(r-1)+10$ and $1 \leq r \leq p/10$ and the other off-diagonal entries of 0. The diagonal entries of Ω' are $\omega_{ii} = 1$ for $i = 1,2,3\ldots,p$. To make the matrix positive definite, the final precision matrix is $\Omega = \Omega' + (|\lambda_{min}| + 0.05)I_p$, where λ_{min} is the minimum eigenvalue of Ω' and I_p is a p by p identity matrix. For $p/10$ variables or nodes in the graph, the expected node degree is 10.
- **E-R graph:** an initial p by p matrix $\Omega' = (\omega_{ij})_{p\times p}$ with each off-diagonal entry $\omega_{ij} = \omega_{ji} = \mu_{ij} * \varphi_{ij}$, where μ_{ij} is a uniform random variable between 0.4 and 0.8 and φ_{ij} is a Bernoulli random variable (1 means success and 0 means failure) with the success probability of $\min(0.05, 5/p)$. The diagonal entries of Ω' are $\omega_{ii} = 1$ for $i = 1,2,3\ldots,p$. To make the matrix positive definite, the final precision matrix is $\Omega = \Omega' + (|\lambda_{min}| + 0.05)I_p$, where λ_{min} is the minimum eigenvalue of Ω' and I_p is a p by p identity matrix. The expected node degree is 5

if $p \geq 100$; otherwise, it is $0.05p$.

2. The graph setting for time evaluation on the implementation of B_NW_SL

 - **E-R graph:** an initial p by p matrix $\Omega' = (\omega_{ij})_{p \times p}$ with each off-diagonal entry $\omega_{ij} = \omega_{ji} =$ a value randomly picked from the set $\{0.3, 0.6, 1\}$, where the probability of each $\omega_{ij} = \omega_{ji} \neq 0$ is π. The diagonal entries of Ω' are all set as 4. Then, all the elements including the diagonals in the bottom right block with a size of $p/2 \times p/2$ in Ω' are multiplied by 2. The final precision matrix is now denoted as Ω. The expected node degree of the graph is πp.
