# Supplementary material for: SILGGM: An extensive R package for efficient statistical inference in large-scale gene networks
Source: PLoS Comput Biol. 2018 Aug 13;14(8):e1006369. doi: 10.1371/journal.pcbi.1006369 (PMC6107288; doi:10.1371/journal.pcbi.1006369)
Supplement: S2 File — (PDF) [file pcbi.1006369.s005.pdf]

S2 File. Testing on the accuracy of individual inference

In this supplementary file, we present the details of evaluation on the accuracy of individual inference of each ω_{ij} (or gene pair) for the cases with $n = 800,\ p = 5000$ and $n = 800,\ p = 10000$. We consider three graph settings described as below:

- **Band graph:** a p by p precision matrix $\Omega = (\omega_{ij})_{p\times p}$ with $\omega_{i,i+1} = \omega_{i+1,i} = 0.6,\ \omega_{i,i+2} = \omega_{i+2,i} = 0.3$ and the other off-diagonal elements $\omega_{ij} = 0$ for $|i - j| \geq 3$. The diagonal entries of Ω are $\omega_{ii} = 1$ for $i = 1,2,3 \ldots, p$. The expected node degree of the graph is 4.
- **E-R graph:** we start with an initial p by p matrix $\Omega' = (\omega_{ij})_{p\times p}$ with each off-diagonal entry $\omega_{ij} = \omega_{ji} = \mu_{ij} * \varphi_{ij}$, where μ_{ij} is a uniform random variable between 0.4 and 0.8 and φ_{ij} is a Bernoulli random variable (1 means success and 0 means failure) with the success probability of $\min(0.05, 5/p)$. The diagonal entries of Ω' are $\omega_{ii} = 1$ for $i = 1,2,3 \ldots, p$. To make the matrix positive definite, the final precision matrix is $\Omega = \Omega' + (|\lambda_{min}| + 0.05)I_p$, where λ_{min} is the minimum eigenvalue of Ω' and I_p is a p by p identity matrix. The expected node degree of the graph is 5 for $p = 5000$ or 10000.
- **Scale-free graph:** By using the preferential attachment scheme, we start with a single node (or gene) and no edges in the first time step. Then, in each time step, a new gene is added, and the newly-added gene initiates an edge to one of the old genes. An old gene i is selected based on the probability $p(i) \propto d(i)^{0.01} + 1$, where $d(i)$ is the node degree of gene

i in the current time step and 0.01 is the power of the preferential attachment. Therefore, the total number of edges in the entire generated graph is given by $p-1$. The above procedure is achieved by the implementation of the function barabasi.game() in the R package igraph. Therefore, we generate a p by p adjacency matrix $\mathrm{A} = (a_{ij})_{p\times p}$ with each off-diagonal element $a_{ij} = 1$ if there is a non-zero partial correlation between gene i and j; otherwise, $a_{ij} = 0$. The diagonal elements of A are all equal to 0. Then, we generate an initial p by p matrix $\Omega' = (\omega_{ij})_{p\times p}$ and set any off-diagonal element $\omega_{ij} = 0.3$ if its corresponding $a_{ij} = 1$. To make the matrix positive definite, the final precision matrix is $\Omega = \Omega' + (|\lambda_{min}| + 0.2)I_p$, where λ_{min} is the minimum eigenvalue of Ω' and I_p is a p by p identity matrix. The following histograms in Fig. A show that the node degree distribution of Scale-free graph for $p = 5000$ and $p = 10000$ follows a power law.

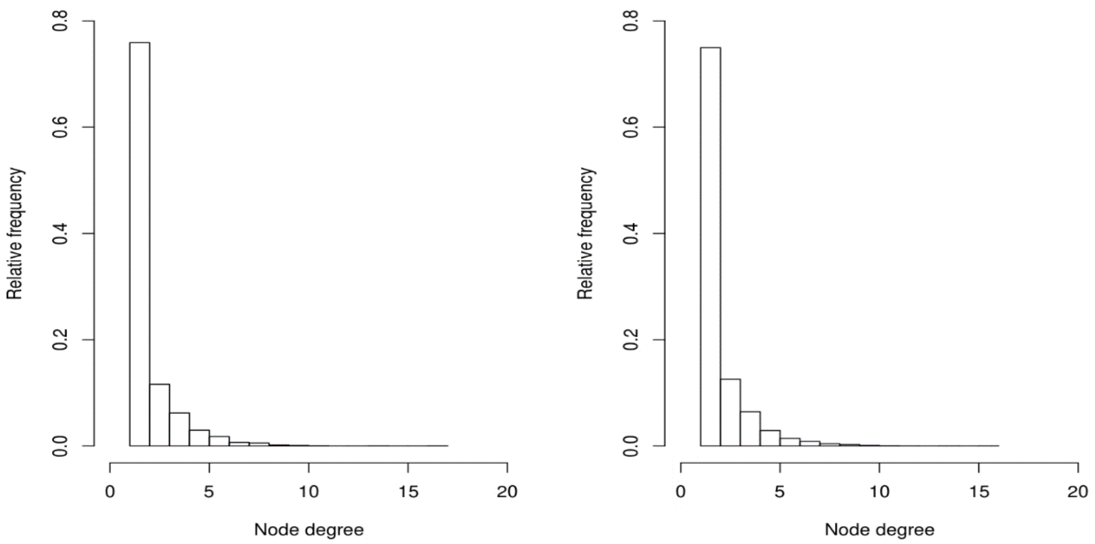

Fig. A. Histograms of node degrees of Scale-free graph. The left plot illustrates the case of $p = 5000$, and the right plot shows the node degree distribution when $p = 10000$.

Under each of the three graph settings, we simulate 100 data sets. We customize GFC_L to be implemented among 5 candidates of tuning parameters for tuning selection, and the other approaches in SILGGM are run with default parameters. We set a pre-specified level of 0.05 on the estimated p-value of each ω_{ij}. In terms of the estimated p-values of all ω_{ij}'s in an entire graph, the mean of the estimated Type I error under the 0.05 level and the corresponding mean of the estimated Type II error over the 100 replications for Band graph, E-R graph and Scale-free graph are reported in the following three tables respectively. The results indicate that all the approaches control the Type I error well in these large scales ($p = 5000/p = 10000$) for individual testing on each gene pair. Also, a non-zero partial correlation can be correctly identified in the case of either $p = 5000$ or $p = 10000$ since the corresponding Type II error for all the simulation settings are around 0.

**Graph setting**	**Average node degree**	p	n	**Methods**	**Type I error (0.05 level)**	**Type II error**
Band	3.9988	5000	800	B_NW_SL	0.0496	0
				D-S_NW_SL	0.0228	0
				D-S_GL	0.0006	0
				GFC_SL	0.0501	0
				GFC_L	0.0503	0
	3.9994	10000	800	B_NW_SL	0.0496	0
				D-S_NW_SL	0.0221	0
				D-S_GL	0.0002	0
				GFC_SL	0.0501	0
				GFC_L	0.0502	0

Graph setting	Average node degree	p	n	Methods	Type I error (0.05 level)	Type II error
E-R	5.0356	5000	800	B_NW_SL	0.0496	9.4×10^{-4}
				D-S_NW_SL	0.0280	1.6×10^{-3}
				D-S_GL	0.0315	8.0×10^{-4}
				GFC_SL	0.0501	9.3×10^{-4}
				GFC_L	0.0501	1.3×10^{-3}
	4.9704	10000	800	B_NW_SL	0.0496	6.0×10^{-4}
				D-S_NW_SL	0.0276	1.1×10^{-3}
				D-S_GL	0.0300	5.6×10^{-4}
				GFC_SL	0.0501	6.0×10^{-4}
				GFC_L	0.0501	8.9×10^{-4}

Graph setting	Average node degree	p	n	Methods	Type I error (0.05 level)	Type II error
Scale-free	1.9996	5000	800	B_NW_SL	0.0495	5.6×10^{-5}
				D-S_NW_SL	0.0427	6.0×10^{-5}
				D-S_GL	0.0415	5.8×10^{-5}
				GFC_SL	0.0501	5.4×10^{-5}
				GFC_L	0.0501	4.8×10^{-5}
	1.9998	10000	800	B_NW_SL	0.0495	7.0×10^{-5}
				D-S_NW_SL	0.0432	8.0×10^{-5}
				D-S_GL	0.0431	8.0×10^{-5}
				GFC_SL	0.0501	7.0×10^{-5}
				GFC_L	0.0501	6.0×10^{-5}

The above validation with Type I and Type II errors for individual inference of whether a known zero or a non-zero partial correlation can be correctly identified based on the information of p-values implies no differences among all the approaches. To make a further comparison for individual inference, we then evaluate the average empirical coverage probabilities for the 95% confidence intervals of the ω_{ij}'s for the "non-zero partial correlation" set S_0 (a set of all pairs with non-zero ω_{ij}'s) and the "zero partial correlation" set ${S_0}^c$ (a set of all pairs with zero ω_{ij}'s) respectively.

Based on the same 100 replications, we report the mean of the 100 estimated average coverage probabilities of the 95% confidence intervals of the ω_{ij}'s in S_0 and the mean of the 100 estimated average coverage probabilities of the 95% confidence intervals of the ω_{ij}'s in ${S_0}^c$ for Band graph, E-R graph and Scale-free graph in the following three tables respectively.

**Graph setting**	**Average node degree**	p	n	**Methods**	S_0	${S_0}^c$
Band	3.9988	5000	800	B_NW_SL	0.9505	0.9504
				D-S_NW_SL	0.7864	0.9772
				D-S_GL	0.5355	0.9994
	3.9994	10000	800	B_NW_SL	0.9496	0.9504
				D-S_NW_SL	0.7368	0.9779
				D-S_GL	0.5538	0.9998

**Graph setting**	**Average node degree**	p	n	**Methods**	S_0	${S_0}^c$
E-R	5.0356	5000	800	B_NW_SL	0.8588	0.9504
				D-S_NW_SL	0.9454	0.9720
				D-S_GL	0.7967	0.9685
	4.9704	10000	800	B_NW_SL	0.8452	0.9504
				D-S_NW_SL	0.9448	0.9724
				D-S_GL	0.7801	0.9700

**Graph setting**	**Average node degree**	p	n	**Methods**	S_0	${S_0}^c$
Scale-free	1.9996	5000	800	B_NW_SL	0.9330	0.9505
				D-S_NW_SL	0.9459	0.9573
				D-S_GL	0.9354	0.9585
	1.9998	10000	800	B_NW_SL	0.9361	0.9505
				D-S_NW_SL	0.9467	0.9568
				D-S_GL	0.9397	0.9569

Since GFC_SL or GFC_L provides no confidence intervals, we involve the other three approaches here. As it can be seen, the results of empirical coverage probabilities in ${S_0}^c$ coincide the ones in Type I error rates and they are all good with our desired level 0.95. For Scale-free graph with $p = 5000$ and 10000, the empirical coverage probabilities of the three methods in S_0 are all around 0.95 as well. However, there are some differences in S_0 for Band graph and E-R graph. For Band graph, B_NW_SL particularly outperforms D-S_NW_SL and D-S_GL since its empirical coverage probabilities in S_0 are well around the desired level, while the empirical coverage probabilities of D-S_NW_SL in S_0 are less than 0.80 and the results of D-S_GL in S_0 are around 0.55. For E-R graph, D-S_NW_SL is the best one with the empirical coverage probabilities in S_0 close to the desired level, but the differences in results among the three methods are much less significant than the ones in Band graph. The empirical coverage probabilities of B_NW_SL in S_0 are still around 0.85, and the results of D-S_GL can be around 0.80 as well.

According to the results from the three graph settings, the overall performance of the confidence intervals among the three methods are good since ${S_0}^c$ is a major part of the sparse graph settings. But in terms of the confidence intervals in S_0 or the non-zero partial correlations, B_NW_SL and D-S_NW_SL perform better than D-S_GL. Moreover, the performance of B_NW_SL is more stable than that of D-S_NW_SL.
