# Supplementary material for: SILGGM: An extensive R package for efficient statistical inference in large-scale gene networks
Source: PLoS Comput Biol. 2018 Aug 13;14(8):e1006369. doi: 10.1371/journal.pcbi.1006369 (PMC6107288; doi:10.1371/journal.pcbi.1006369)
Supplement: S3 File — (PDF) [file pcbi.1006369.s006.pdf]

S3 File. Testing on the accuracy of global inference

In this supplementary file, we show the details of evaluation on the accuracy of global inference which requires a simultaneous testing on all ω_{ij}'s (or gene pairs) with $H_0: \omega_{ij} = 0$ vs. $H_1: \omega_{ij} \neq 0$ for $1 \leq i < j \leq p$. We consider the same three graph settings as shown in S2 File:

- **Band graph:** a p by p precision matrix $\Omega = (\omega_{ij})_{p\times p}$ with $\omega_{i,i+1} = \omega_{i+1,i} = 0.6$, $\omega_{i,i+2} = \omega_{i+2,i} = 0.3$ and the other off-diagonal elements $\omega_{ij} = 0$ for $|i - j| \geq 3$. The diagonal entries of Ω are $\omega_{ii} = 1$ for $i = 1,2,3 \dots, p$. The expected node degree of the graph is 4.
- **E-R graph:** we start with an initial p by p matrix $\Omega' = (\omega_{ij})_{p\times p}$ with each off-diagonal entry $\omega_{ij} = \omega_{ji} = \mu_{ij} * \varphi_{ij}$, where μ_{ij} is a uniform random variable between 0.4 and 0.8 and φ_{ij} is a Bernoulli random variable (1 means success and 0 means failure) with the success probability of $\min(0.05, 5/p)$. The diagonal entries of Ω' are $\omega_{ii} = 1$ for $i = 1,2,3 \dots, p$. To make the matrix positive definite, the final precision matrix is $\Omega = \Omega' + (|\lambda_{min}| + 0.05)I_p$, where λ_{min} is the minimum eigenvalue of Ω' and I_p is a p by p identity matrix. The expected node degree of the graph is 5 for $p = 5000$ or 10000.
- **Scale-free graph:** By using the preferential attachment scheme, we start with a single node (or gene) and no edges in the first time step. Then, in each time step, a new gene is added, and the newly-added gene initiates an edge to one of the old genes. An old gene i is selected based on the

probability $p(i) \propto d(i)^{0.01} + 1$, where $d(i)$ is the node degree of gene i in the current time step and 0.01 is the power of the preferential attachment. Therefore, the total number of edges in the entire generated graph is given by $p - 1$. The above procedure is achieved by the implementation of the function barabasi.game() in the R package igraph. Therefore, we generate a p by p adjacency matrix $\mathrm{A} = (a_{ij})_{p \times p}$ with each off-diagonal element $a_{ij} = 1$ if there is a non-zero partial correlation between gene i and j; otherwise, $a_{ij} = 0$. The diagonal elements of A are all equal to 0. Then, we generate an initial p by p matrix $\Omega' = (\omega_{ij})_{p \times p}$ and set any off-diagonal element $\omega_{ij} = 0.3$ if its corresponding $a_{ij} = 1$. To make the matrix positive definite, the final precision matrix is $\Omega = \Omega' + (|\lambda_{min}| + 0.2)I_p$, where λ_{min} is the minimum eigenvalue of Ω' and I_p is a p by p identity matrix. The following histograms in Fig. A show that the node degree distribution of Scale-free graph for $p = 5000$ and $p = 10000$ follows a power law.

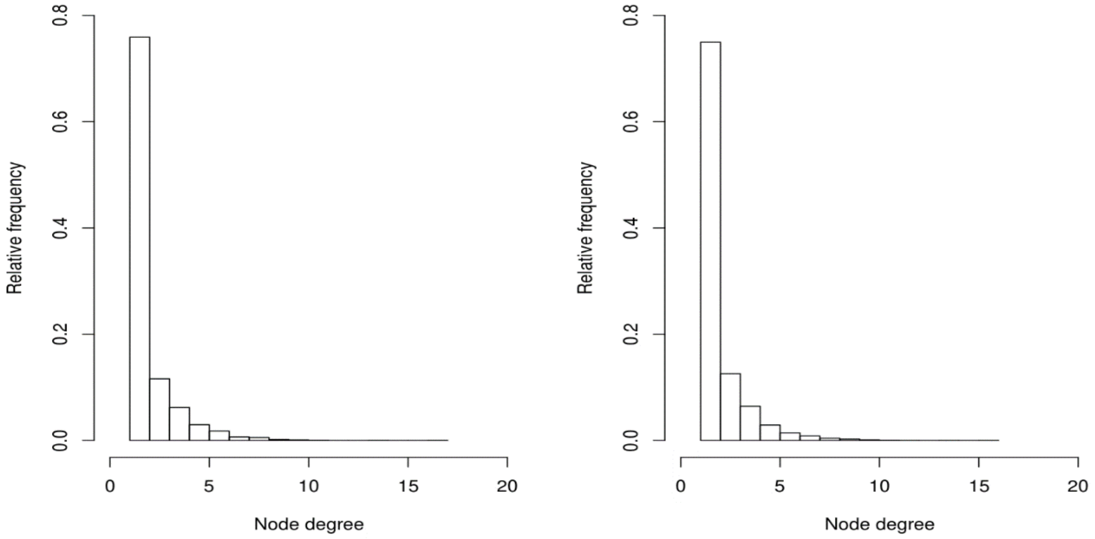

Fig. A. Histograms of node degrees of Scale-free graph. The left plot illustrates the case of $p = 5000$, and the right plot shows the node degree distribution when $p = 10000$.

As for accuracy metrics, we use false discovery rate (FDR), power and the Matthews correlation coefficient (MCC). FDR is the expected proportion of false "discoveries" (the number of incorrect rejections on H_0's) among the total "discoveries" (the total number of rejections on H_0's). We need to control FDR to avoid the inflation of false positives through global inference. The details of the FDR procedure can be referred to S1 Appendix. As the second measure, the corresponding power is to show to what extent that the total number of true non-zero partial correlations can be correctly identified through the FDR procedure. Besides FDR and the corresponding power, we also consider MCC as the third measure. Here, MCC is used to gauge how well the known zero partial correlations and the known non-zero partial correlations can be correctly identified through the FDR procedure. It is well known that MCC is even robust to class imbalances, so it tailors to our sparse graph settings which have far more

the zero partial correlations than the non-zero partial correlations. Note that MCC lies in the interval between -1 and 1. A value of 1 indicates a perfect selection of all the known zero and the non-zero partial correlations, while a value of -1 implies a total disagreement between prediction and the true partial correlations. A value of 0 means a random guess. Therefore, a closer value of MCC to 1 suggests a better identification of the overall zero and non-zero partial correlations.

For each of the three graph settings, we use the same 100 simulated datasets as used in S2 File. When implementing all the approaches, we set the argument `alpha = 0.05` to denote a pre-specified level of 0.05 for FDR control. We further set the argument `global = TRUE` when implementing D-S_NW_SL, D-S_GL and B_NW_SL to include global inference. In addition, we customize GFC_L to be implemented among 5 candidates of tuning parameters for tuning selection. With the 100 simulated data sets and the pre-specified level of FDR set at $\alpha = 0.05$, the empirical FDRs of all the graph settings for $p = 5000$ and $p = 10000$, the corresponding mean power values and the corresponding average MCCs are reported in the following three tables.

**Graph setting**	**Average node degree**	p	n	**Methods**	**FDR (0.05 level)**	**Power**	**MCC**
Band	3.9988	5000	800	B_NW_SL	0.0386	1.0000	0.9805
				D-S_NW_SL	0.0024	1.0000	0.9988
				D-S_GL	0.0098	1.0000	0.9951
				GFC_SL	0.0365	1.0000	0.9816
				GFC_L	0.0470	1.0000	0.9762
	3.9994	10000	800	B_NW_SL	0.0370	1.0000	0.9813
				D-S_NW_SL	0.0021	1.0000	0.9989
				D-S_GL	0.0139	0.9993	0.9927
				GFC_SL	0.0362	1.0000	0.9817
				GFC_L	0.0466	1.0000	0.9764

Graph setting	Average node degree	p	n	Methods	FDR (0.05 level)	Power	MCC
E-R	5.0356	5000	800	B_NW_SL	0.0385	0.9236	0.9423
				D-S_NW_SL	0.0093	0.8941	0.9411
				D-S_GL	0.0149	0.9199	0.9519
				GFC_SL	0.0371	0.9156	0.9389
				GFC_L	0.0328	0.9014	0.9337
	4.9704	10000	800	B_NW_SL	0.0371	0.9211	0.9417
				D-S_NW_SL	0.0078	0.8874	0.9383
				D-S_GL	0.0122	0.9105	0.9483
				GFC_SL	0.0371	0.9129	0.9375
				GFC_L	0.0325	0.8962	0.9311

Graph setting	Average node degree	p	n	Methods	FDR (0.05 level)	Power	MCC
Scale-free	1.9996	5000	800	B_NW_SL	0.0361	0.9367	0.9502
				D-S_NW_SL	0.0296	0.9334	0.9517
				D-S_GL	0.0234	0.9310	0.9535
				GFC_SL	0.0438	0.9424	0.9493
				GFC_L	0.0454	0.9472	0.9509
	1.9998	10000	800	B_NW_SL	0.0341	0.8809	0.9224
				D-S_NW_SL	0.0293	0.8761	0.9222
				D-S_GL	0.0251	0.8739	0.9230
				GFC_SL	0.0445	0.8929	0.9237
				GFC_L	0.0454	0.8986	0.9262

As we can see, the FDRs of all the methods for the three graph settings are effectively controlled below the desired 0.05 level for both $p = 5000$ and $p = 10000$. In terms of Band graph, almost all the power values are 1 such that the non-zero partial correlations can be correctly identified under the well-controlled FDRs. MCCs of all the methods are close to 1, indicating a near-perfect identification of the overall zero and non-zero partial correlations. For E-R graph, all the approaches have comparable results of power and MCC, and the performance of B_NW_SL is slightly

better considering both power and MCC. Similarly, the results of power and MCC of all the methods are also very close for Scale-free graph, and the performance of GFC_L is slightly better according to power and MCC. Even though the overall results are slightly worse for E-R graph and Scale-free graph due to their far more randomized structures than Band graph, all the methods still show high values of power and MCC even in the cases of $p = 10000$. When $p = 10000$, all the power values are around 0.90, and the MCCs are about 0.95 for E-R graph. For Scale-free graph, the power values of all the methods are almost 0.90, and the MCCs are still more than 0.92. Among the three graph settings, even though the FDRs of D-S_NW_SL and D-S_GL are controlled more conservatively below the desired level, their power values and MCCs do not suffer a noticeably negative impact, and some results are even better compared to the other approaches in some particular settings. Therefore, all the approaches have shown good performance in correctly identifying the zero and the non-zero partial correlations in a global sense even for the very high-dimensional scenarios.

In addition, we have also clarified the mean numbers of false positives (incorrect rejections on the true H_0's) of each approach and the corresponding mean false positive rates (the proportions of false positives among the true H_0's) in the previous benchmarking with the FDR procedure in the following three tables based on the 100 replications. We can see from the tables that all the false positive rates are close to 0. In other words, the observed false positive numbers are acceptable given the huge number of true negatives (true H_0's).

**Graph setting**	**Average node degree**	$\boldsymbol{p}$	$\boldsymbol{n}$	**Methods**	**Number of false positives**	**False positive rate**
Band	3.9988	5000	800	B_NW_SL	401.8	3.2×10^{-5}
				D-S_NW_SL	24.1	1.9×10^{-6}
				D-S_GL	98.7	7.9×10^{-6}
				GFC_SL	378.7	3.0×10^{-5}
				GFC_L	493.3	4.0×10^{-5}
	3.9994	10000	800	B_NW_SL	768.7	1.5×10^{-5}
				D-S_NW_SL	42.3	8.5×10^{-7}
				D-S_GL	282.5	5.7×10^{-6}
				GFC_SL	751.8	1.5×10^{-5}
				GFC_L	976.5	2.0×10^{-5}

**Graph setting**	**Average node degree**	$\boldsymbol{p}$	$\boldsymbol{n}$	**Methods**	**Number of false positives**	**False positive rate**
E-R	5.0356	5000	800	B_NW_SL	466.1	3.7×10^{-5}
				D-S_NW_SL	105.3	8.4×10^{-6}
				D-S_GL	175.0	1.4×10^{-5}
				GFC_SL	443.6	3.6×10^{-5}
				GFC_L	384.6	3.1×10^{-5}
	4.9704	10000	800	B_NW_SL	882.1	1.8×10^{-5}
				D-S_NW_SL	172.7	3.5×10^{-6}
				D-S_GL	278.7	5.6×10^{-6}
				GFC_SL	875.0	1.8×10^{-5}
				GFC_L	748.9	1.5×10^{-5}

**Graph setting**	**Average node degree**	$\boldsymbol{p}$	$\boldsymbol{n}$	**Methods**	**Number of false positives**	**False positive rate**
Scale-free	1.9996	5000	800	B_NW_SL	175.5	1.4×10^{-5}
				D-S_NW_SL	142.2	1.1×10^{-5}
				D-S_GL	111.6	9.0×10^{-6}
				GFC_SL	215.7	1.7×10^{-5}
				GFC_L	225.1	1.8×10^{-5}
	1.9998	10000	800	B_NW_SL	311.4	6.3×10^{-6}
				D-S_NW_SL	264.4	5.3×10^{-6}
				D-S_GL	225.1	4.5×10^{-6}
				GFC_SL	415.6	8.3×10^{-6}
				GFC_L	427.4	8.6×10^{-6}
