# Supplementary material for: SILGGM: An extensive R package for efficient statistical inference in large-scale gene networks
Source: PLoS Comput Biol. 2018 Aug 13;14(8):e1006369. doi: 10.1371/journal.pcbi.1006369 (PMC6107288; doi:10.1371/journal.pcbi.1006369)
Supplement: S1 Table — (PDF) [file pcbi.1006369.s007.pdf]

Table S1. Timings (in seconds) of B_NW_SL (SILGGM) and B_NW_SL (FastGGM)

**Average node degree**	$\boldsymbol{\pi}$	$\boldsymbol{p}$	$\boldsymbol{n}$	**B_NW_SL (SILGGM)**	**B_NW_SL (FastGGM)**
4.045	0.005	800	400	24.9	36.6
4.994	0.005	1000	800	72.8	145.2
4.970	0.0025	2000	800	411.7	938.5
5.0264	0.001	5000	800	5772.6	8663.7
5.0498	0.0005	10000	800	40080.6	49650.2
